# Skill Proficiency, Efficacy, and Safety of the Transradial Approach in Transarterial Treatments for Hepatocellular Carcinoma

**DOI:** 10.7759/cureus.57800

**Published:** 2024-04-08

**Authors:** Kyo Sasaki, Yusuke Kawamura, Chikara Ogawa, Kazuhisa Yabushita, Shoji Watanabe, Hideki Hayashi, Takahiro Kochi, Tetsu Tomonari, Masashi Ninomiya, Koji Takai, Kento Imajo, Takanori Ito, Ryuichi Kita, Seiichi Mawatari, Sohji Nishina, Hidenori Toyoda

**Affiliations:** 1 Department of Gastroenterology, Kawasaki Medical School, Kurashiki, JPN; 2 Department of Hepatology, Toranomon Hospital, Tokyo, JPN; 3 Department of Gastroenterology and Hepatology, Takamatsu Red Cross Hospital, Takamatsu, JPN; 4 Division of Internal Medicine, Fukuyama City Hospital, Fukuyama, JPN; 5 Department of Gastroenterology, JA Niigata Kouseiren Ojiya General Hospital, Ojiya, JPN; 6 Department of Gastroenterology and Hepatology, Gifu Municipal Hospital, Gifu, JPN; 7 Department of Gastroenterology and Oncology, Tokushima University Graduate School of Biomedical Sciences, Tokushima, JPN; 8 Division of Gastroenterology, Tohoku University Graduate School of Medicine, Sendai, JPN; 9 Department of Gastroenterology/Internal Medicine, Gifu University Graduate School of Medicine, Gifu, JPN; 10 Department of Gastroenterology and Hepatology, Yokohama City University Graduate School of Medicine, Yokohama, JPN; 11 Department of Gastroenterology and Hepatology, Nagoya University Hospital, Nagoya, JPN; 12 Department of Gastroenterology and Hepatology, Osaka Red Cross Hospital, Osaka, JPN; 13 Department of Human and Environmental Sciences, Kagoshima University Graduate School of Medical and Dental Sciences, Kagoshima, JPN; 14 Department of Gastroenterology, Ogaki Municipal Hospital, Ogaki, JPN

**Keywords:** transradial access, hepatocellular carcinoma, hepatic angiography, comfort, clinical benefit

## Abstract

Introduction

Abdominal angiography procedures such as transarterial chemoembolization (TACE) are essential for hepatocellular carcinoma treatment. One method commonly used is transfemoral access (TFA). However, issues associated with this method, which include postoperative compression of the puncture site and long periods of bed rest, can affect patient satisfaction. Thus, transradial access (TRA), a minimally invasive treatment method that improves treatment quality, was developed for TACE. This retrospective, multicenter study aimed to investigate the efficacy and safety of abdominal angiography using the radial artery approach.

Methods

In total, 1,601 patients underwent abdominal angiography using TRA and received treatment (radial access for visceral intervention (RAVI)) at 14 institutions in Japan. The treatment time, procedure completion rate, patient satisfaction, and complications were investigated.

Results

The success rate of RAVI was 99.4%, and the complication rate was 1.2%. Approximately 98.2% of the patients requested the radial artery approach again. There were no significant differences in the success rate of RAVI and the incidence of complications based on the operator's years of experience or the patient’s age. Some patients developed minor complications such as puncture site bleeding, hematoma, vascular pain, and vasospasm. Further, serious complications (cerebral infarction (n = 1), cerebellar infarction (n = 1), and aortic dissection (n = 1)) were observed.

Conclusion

Similar to the conventional TFA, RAVI helped in facilitating peritoneal angiography safely. In abdominal angiography, this method can reduce patient burden and can be widely used in the future from the perspective of clinical benefit.

## Introduction

Transcatheter hepatic artery chemoembolization (TACE) and hepatic arteriography are important techniques that are indispensable for hepatocellular carcinoma (HCC) treatment. They are recommended for unresectable HCC based on the liver cancer treatment guidelines of every country and are recognized as one [1,2]. Traditionally, transfemoral access (TFA) has been the primary method in TACE for HCC. Meanwhile, in the cardiovascular field, angiography is commonly performed using transradial access (TRA), which has a similar or higher performance than TFA.

Some reports have shown that complications can be reduced with treatment [3]. In several countries, some facilities perform TRA for abdominal angiography. Furthermore, compared with TFA, TRA has better outcomes in liver cancer treatment, is associated with a lower incidence of complications and a shorter hospital stay, and has medical economic advantages [4,5]. Recently, in Japan, TRA has been found to be safe and useful [6-8]. In addition to improving treatment efficacy, minimally invasive treatment is an important element in light of a recent medical trend. Moreover, it should be considered in future HCC treatment as it can reduce the need for postoperative rest. The current study aimed to evaluate the tolerability, efficacy, and safety of the radial artery catheter approach, which is becoming more widespread at various facilities across the world.

## Materials and methods

Participants

This was a multicenter retrospective study that collected and retrospectively analyzed data from 14 facilities in Japan that have introduced the radial artery approach examining and treating liver tumors by a physician with experience in TACE using the femoral approach. A total of 1,601 patients who underwent radial artery approach angiography and treatment for liver tumor investigation and treatment at participating institutions between January 1, 2008, and March 31, 2023, were enrolled. In this study, cases diagnosed with HCC through imaging tests such as CT and MRI were included, and cases with missing data were excluded. We investigated the characteristics of patients who underwent radial access for visceral intervention (RAVI), duration of RAVI, years of operator experience, success rate of treatment and testing, patient satisfaction, and complications. The study protocol was in accordance with the Declaration of Helsinki and was approved by the Ethics Committee of Ogaki Municipal Hospital (approval number: 20230323-5). The need for informed consent was waived because only de-identified data collected from medical records were used.

Hepatic angiography procedures with access through the radial artery

Tests and treatments were performed at each facility using angiography equipment. A positive Allen's test was performed before surgery. Some facilities performed ultrasonography to check for carotid artery plaque and CT scans to evaluate for calcification and the shape of the aorta before surgery. Local anesthetic using lidocaine tape was applied to the left radial puncture site 30 minutes before surgery. This reduced the amount of local anesthesia and made it easier to puncture the blood vessels.

**Figure 1:**
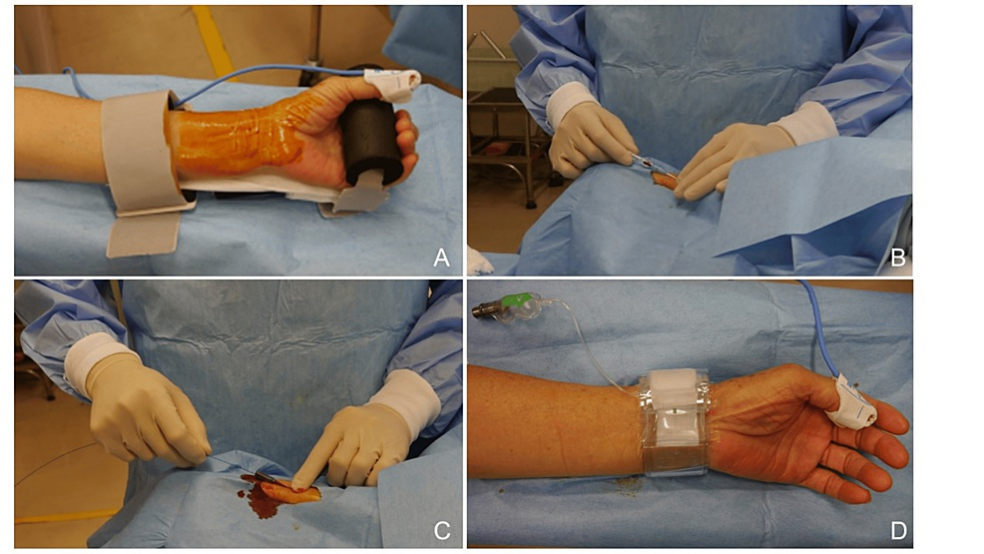
The left radial artery puncture technique (A) A lidocaine tape was applied to the puncture site before the procedure, and the same area was disinfected. (B) Puncturing was performed with a 22-G Seldinger needle. (C) A 4-Fr sheath was secured under guidewire guidance. (D) The puncture site was compressed with a TR band.

A typical procedure flow in this cohort is described below. The patient was placed in the supine position, and the area around the left radial artery was disinfected. After administering local anesthesia with 1% lidocaine, the left radial artery was punctured with a 22-G Seldinger needle, and a 4-Fr sheath (Radifocus™ Introducer II, RR-AF4J16H, 4 Fr, 16 cm; Terumo Corporation, Tokyo, Japan) was placed under inserter guidance (Figure 1A-1C). Some facilities perform punctures while observing the radial artery under ultrasound guidance.

**Figure 2:**
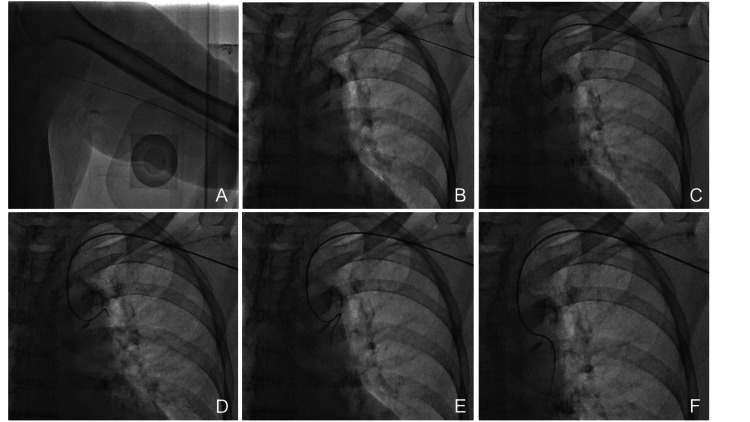
Introduction of a catheter into the descending aorta (A–C) The catheter was cautiously passed through the left subclavian artery under guidewire guidance. Then, it was advanced to the aortic arch. (D–F) The guidewire was adequately inserted into the descending aorta, and it guided the catheter. Based on the shape of the aortic arch, a pigtail catheter was used.

After sheath placement, 2000 units of heparin sodium were intravenously injected to prevent cerebrovascular embolism, and 1000 units were added every hour during the surgery. Under the guidance of a guidewire (Radifocus Guide Wire M, RF-GA 35153, 0.035 inch, 150 cm; Terumo Corporation), the abdominal angiography catheter (Sakaguchi SG-42-120S2-U4,4 Fr, 120 cm; Gadelius Holding Ltd., Tokyo, Japan) or (Radifocus Glidecath Ⅱ, Tip shape RAVI MG1, 5 Fr, 125 cm; Terumo Corporation) was inserted into the descending aorta, being careful not to stray into the vertebral artery (Figure 2A-2F). In cases where it is anatomically difficult to cannulate the descending aorta from the aortic arch or if aortography should be performed initially, a pigtail catheter (Straight Pigtail, RQ-4SP0081M, 4 Fr, 110 cm; Terumo Corporation) was used to cannulate the descending aorta [7]. Descending aorta cannulation was performed by tilting the radiographic equipment (LAO of 15°) or via deep inspiration. However, from the perspective of preventing complications, it is important not to spend too much time investigating the aortic arch by changing surgeons or using a pigtail catheter. To insert a catheter into the celiac artery or superior mesenteric artery, the catheter was hooked into the target blood vessel by rotating it clockwise at the level of the blood vessel bifurcation. The subsequent procedures varied per institution; however, standard angiography and treatment were performed. The length of the microcatheter must be 150 cm.

When removing the catheter, the presence of any residual thrombus inside the catheter must be evaluated. Then, the guide wire should be placed inside the catheter and removed with the tip fully extended. When removing the sheath, the TR band (TR Band™ XX-RF06; Terumo Corporation) must be wrapped around the puncture site of the left radial artery, pressure (13 ml) should be applied, and the sheath must be removed. The pressure was then reduced over time and removed the next morning. In the case of rebleeding, the TR band was repressurized to 13 ml (Figure 1D).

Evaluation via head MRI

Head MRI was performed within three days after RAVI to evaluate the presence of lacunar infarctions. The current study included eight patients at two facilities, and the images were interpreted and evaluated by two radiological specialists.

Statistical analysis

Categorical variables were expressed as numbers and percentages and continuous variables as medians and interquartile ranges. Differences in percentages between groups were analyzed using the chi-square test. All P values were derived from two-tailed tests, and a P value of <0.05 was considered statistically significant.

## Results

Characteristics of the patients

Table 1 shows the characteristics of the patients. The median age of the patients was 74 years, and approximately 76.7% were men. Further, 48.1% of cases were non-viral. In total, 1049 (65.5%) patients presented with cardiovascular risk factors, and 261 (16.3%) had a history of cardiovascular events. The median number of years of TACE experience was 17 years, and RAVI was performed on 1297 (79.1%) patients for therapeutic purposes and 334 (20.9%) for diagnostic purposes. The median total treatment and examination time was 85 minutes, and the median time from radial artery sheath placement to the first contrast-enhanced imaging was 17 minutes. Pigtail catheters were used for cannulation of the descending aorta or aortography in 26.5% of cases, with a procedural success rate of 99.4% and a complication rate of 1.6%. The median length of hospital stay was six days. In a questionnaire survey of patients regarding the next puncture site, 728 (98.2%) of 741 preferred the radial artery approach again.　

**Table 1:** Characteristics of the patients and treatment details for all patients undergoing RAVI for HCC *Note*: †Data were presented as case number (percentage). ‡Data were expressed as median (range). §At least one cardiovascular risk factor, including current history of smoking, hypertension, hypercholesterolemia, diabetes mellitus, obesity or overweight, physical inactivity, and alcohol consumption (>10 standard drinks per week or >4 standard drinks per day). HBV, hepatitis B virus; HCV, hepatitis C virus; ALBI, albumin-bilirubin; TACE, transcatheter arterial chemoembolization; RAVI, radial access for visceral intervention; HCC, hepatocellular carcinoma

	All patients (N = 1601)
Age, years, median (range) ‡	74 (34–94)
Sex, female/male, n (%) †	369 (23.3%)/1228 (76.7%)
Etiology: HBV/HCV/non-viral, n (%) †	636 (51.9%)/589 (48.1%)
Platelet count, 104/μL, median (range) ‡	13.1 (0.4–45.2)
Albumin level, g/dL, median (range) ‡	3.7 (1.8–5.2)
Bilirubin level, mg/dL, median (range) ‡	0.9 (0.1–8.0)
ALBI score, median (range) ‡	-2.39 (-3.87 to 0.65)
Prothrombin time, median (range) ‡	85.0 (17–150)
Procedure: arteriography alone/TACE, n (%) †	334 (20.9%) / 1297 (79.1%)
Years of TACE experience for practitioners, years, median (range) ‡	17 (1–33)
History of cardiovascular events, no/yes, n (%) †	1340 (83.7%) / 261 (16.3%)
Cardiovascular risk factors§, no/yes, n (%) †	552 (34.5%) / 1049 (65.5%)
Total procedure time, minutes, median (range) ‡	85 (23–358)
Time from sheath placement to the first contrast imaging, minutes, median (range) ‡	17 (2–120)
Use of pigtail catheters, no/yes, n (%) †	1177 (73.5%) / 424 (26.5%)
Successful procedure, no/yes, n (%) †	10(0.6%) / 1589 (99.4%)
Complications, no/yes, n (%) †	1576 (98.4%) / 25 (1.6%)
Median length of hospitalization, days, median (range) ‡	6 (1–44)
Radius as the desired puncture site for the next procedure, no/yes, n (%) †	13 (1.8%) / 728 (98.2%)

Comparison according to years of RAVI experience and patient age

We compared the incidence of complications and treatment effects in patients with RAVI experience of <1 year (n = 372) and ≥1 (n = 1299). In terms of the characteristics of the patients, there were significant differences in the purpose of RAVI and the history of cardiovascular events. There were no significant differences in terms of treatment time. However, patients with a RAVI experience of >1 year had a significantly lower procedural success rate and incidence of complications and a shorter length of hospital stay and time from sheath placement to the first contrast imaging (Table 2). A comparison of patients aged <80 years (n = 1174) and those aged >80 years (n = 422) revealed significant differences in terms of age, sex, purpose of RAVI, prevalence of cardiovascular risk factors, and history of cardiovascular events. However, no differences were observed in the treatment time, procedural success rate, length of hospital stay, and complication rate (Table 3).

**Table 2:** Comparison based on the years of RAVI experience of practitioners (<1 vs. ≥1 year of experience) *Note*: †Data were presented as case number (percentage). ‡Data were expressed as median (range). §At least one cardiovascular risk factor, including current history of smoking, hypertension, hypercholesterolemia, diabetes mellitus, obesity or overweight, physical inactivity, and alcohol consumption (>10 standard drinks per week or >4 standard drinks per day). HBV, hepatitis B virus; HCV, hepatitis C virus; ALBI, albumin-bilirubin; TACE, transcatheter arterial chemoembolization; RAVI, radial access for visceral intervention

	Practitioners with <1 year of experience (N=372)	Practitioners with>1 year of experience (N=1229)	P value
Age, years, median (range) ^‡^	74 (36–92)	74 (34–94)	n.s
Sex, female/male, n (%) ^†^	79 (21.2%)/293 (78.8%)	291 (23.7%)/934 (76.0%)	n.s
Etiology, HBV/HCV/non-viral, n (%)^†^	189 (50.8%)/183 (49.2%)	448 (52.5%)/405 (47.5%)	n.s
Platelet count,10^4^/μL, median (range) ^‡^	13.1 (0.4–45.2)	13.0 (2.2–40.2)	n.s
Albumin level, g/dL, median (range) ^‡^	3.7 (2.0–5.1)	3.7 (1.8–5.2)	n.s
Bilirubin level, mg/dL, median (range) ^‡^	0.9 (0.2–7.0)	0.9 (0.1-8.0)	n.s
ALBI score, median (range) ^‡^	−2.35 (−3.49 to 0.78)	−2.42 (−3.87 to 0.65)	n.s
Prothrombin time, median (range) ^‡^	86.0 (17–150)	85.0 (21–150)	n.s
Procedure: arteriography alone/TACE, n (%) ^†^	61 (16.4%)/311 (83.6%)	273 (22.2%)/956 (77.8%)	0.02
History of cardiovascular events, no/yes, n (%) ^†^	291 (78.2%)/81 (21.8%)	1050 (85.4%)/179 (14.6%)	<0.001
Cardiovascular risk factors^§^, no/yes, n (%) ^†^	116 (31.2%)/256 (68.8%)	437 (35.5%)/792 (64.4%)	n.s.
Total procedure time, minutes, median (range) ^‡^	86 (24–260)	85 (23–358)	n.s
Time from sheath placement to the first contrast imaging, minutes, median (range)^‡^	25 (4–99)	17 (3–120)	<0.0001
Use of pigtail catheters, no/yes, n (%) ^†^	261 (70.2%)/111(29.8%)	916 (74.5%)/313 (25.5%)	n.s
Successful procedure, no/yes, n (%) ^†^	2 (0.5%)/368 (99.5%)	8 (0.7%)/1221 (99.3%)	n.s
Complications, no/yes, n (%) ^†^	362 (97.3%)/10 (2.7%)	1217 (98.8%)/15 (1.2%)	n.s
Median length of hospitalization, days, median (range) ^‡^	5 (2–44)	6 (1–38)	n.s.
Radius as the desired puncture site for the next procedure, no/yes, n (%)^†^	7 (2.9%)/231 (97.1%)	5 (1.0%)/497 (99.0%)	n.s

**Table 3:** Comparison according to age (<80 vs. ≥80 years) *Note*: †Data were expressed as case number (percentage). ‡Data were expressed as median (range). §At least one cardiovascular risk factor, including current history of smoking, hypertension, hypercholesterolemia, diabetes mellitus, obesity or overweight, physical inactivity, and alcohol consumption (>10 standard drinks per week or >4 standard drinks per day). HBV, hepatitis B virus; HCV, hepatitis C virus; ALBI, albumin-bilirubin; TACE, transcatheter arterial chemoembolization

	Practitioners with <1 year of experience (n=1174)	Practitioners with>1 year of experience (N=422)	P value
Age, years, median (range) ^‡^	71 (34–79)	83 (80–94)	<0.0001
Sex, female/male, n (%) ^†^	239 (20.4%)/931 (79.6%)	129 (30.6%)/293 (69.4%)	<0.001
Etiology, HBV/HCV/non-viral, n (%)^ †^	452 (51.4%)/427(48.6%)	181 (53.1%)/160 (46.9%)	n.s
Platelet count,10^4^/μL, median (range) ^‡^	12.6 (2.2–45.2)	14.0 (0.4–35.6)	n.s
Albumin level, g/dL, median (range) ^‡^	3.7 (2.0–5.2)	3.7 (1.8–4.8)	n.s
Bilirubin level, mg/dL, median (range) ^‡^	0.9 (0.1–8.0)	0.8 (0.1–3.2)	n.s
ALBI score, median (range) ^‡^	−2.39 (−3.87 to 0.65)	−2.42 (−3.46 to 0.91)	n.s
Prothrombin time, median (range) ^‡^	85 (23–150)	87 (17–144.8)	n.s
Procedure: arteriography alone/TACE, n (%) ^†^	260 (22.1%)/914(77.9%)	72 (17.1%)/352 (82.9%)	0.02
History of cardiovascular events, no/yes, n (%) ^†^	1018 (86.9%)/154(13.1%)	316 (74.9%)/106 (25.1%)	<0.001
Cardiovascular risk factors^§^, no/yes, n (%) ^†^	436 (37.1%)/738 (62.9%)	115 (27.3%)/307 (72.7%)	<0.001
Total procedure time, minutes, median (range) ^‡^	85 (23–358)	87 (23–260)	n.s
Time from sheath placement to the first contrast imaging, minutes, median (range)^‡^	18 (3–104)	20 (4–120)	n.s
Use of pigtail catheters, no/yes, n (%) ^†^	854 (72.7%)/320 (27.3%)	322 (76.3%)/100 (23.7%)	n.s
Successful procedure, no/yes, n (%) ^†^	6 (0.5%)/1167 (99.5%)	4 (0.9%)/417 (99.1%)	n.s
Complications, no/yes, n (%) ^†^	1156 (98.5%)/18 (1.5%)	415 (98.3%)/7 (1.7%)	n.s
Median length of hospitalization, days, median (range)^‡^	6 (1–38)	7 (2–44)	n.s.
Radius as the desired puncture site for the next procedure, no/yes, n (%)^†^	10 (1.9%)/516 (98.1%)	2 (0.9%)/210 (99.1%)	n.s

Complications of RAVI

In total, 25 (1.2%) patients developed complications specific to the radial artery approach. Moreover, 12 patients experienced rebleeding at the radial puncture site. However, hemostasis was achieved in all patients by reapplying pressure with the TR band. In addition, hematoma formation was observed in six cases, radial artery spasm in two, and radial nerve pain in two. However, there were no cases with residual sequelae. Some patients developed serious complications but all recovered with conservative treatment (cerebral infarction (n = 1), cerebellar infarction (n = 1), and aortic dissection (n = 1)) (Table 4).

**Table 4:** Safety outcomes and related complication of RAVI (N=25, 1.6%) *Note*: Data were expressed as number (percentage). RAVI, radial access for visceral intervention

RAVI complications	n (%)
Radial puncture site bleeding	12 (0.7%)
Hematoma	6 (0.4%)
Radial artery spasm	2 (0.1%)
Radial pain and numbness	2 (0.1%)
Pseudoaneurysm	0 (0%)
Aorta dissection	1 (0.1%)
Cerebral infraction	2 (0.1%)

RAVI complicated by cerebellar infarction

**Figure 3:**
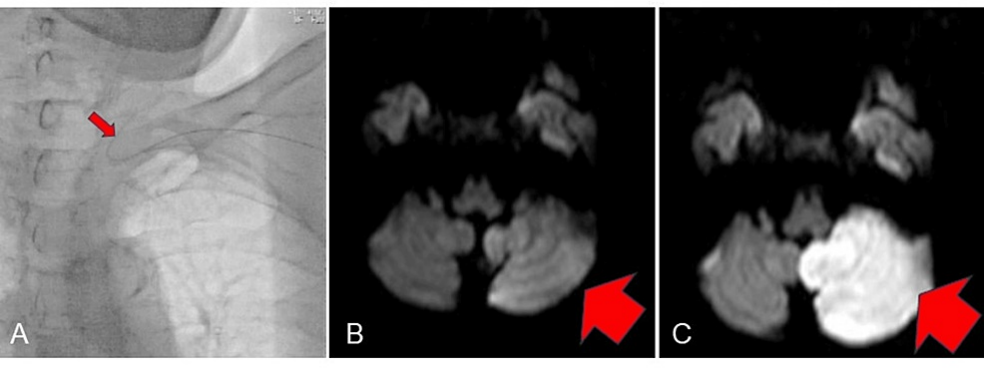
Case of left cerebellar infarction caused by RAVI (A) A guide wire was inadvertently inserted into the left vertebral artery. (B) MRI (DWI) was performed immediately after onset.  (C) MRI (DWI) was performed on the day after onset. RAVI, radial access for visceral intervention; DWI, diffusion-weighted imaging

A male patient in his 70s underwent left and right femoral artery bypass surgery for arteriosclerosis obliterans in his right lower extremity. RAVI-TACE was performed on HCC in segments 5/8 and 7 of the liver. The guide wire strayed into the left vertebral artery while passing through the left subclavian artery (Figure 3A). The patient complained of feelings of floating, and a head MRI revealed hyperintense changes in the left cerebellar hemisphere on diffusion-weighted imaging (DWI) (Figure 3B). He was then diagnosed with left cerebellar infarction, and treatment with tissue plasminogen activator (t-PA) was started. On the next day, an MRI was performed, and results showed extensive infarction in the left cerebellum on DWI, and left cerebellar ataxia remained. However, the patient was discharged home on the 41st day of onset (Figure 3C). Head MRI was performed within three days of RAVI to confirm whether lacunar infarction was caused by RAVI. Eight patients underwent head MRI at two facilities, and no new cerebral infarctions, including lacunar infarctions, were detected.

## Discussion

With recent advancements in drug therapy for hepatocellular carcinoma, the therapeutic indications for TACE are changing, and more selective TACE is required. In addition, minimally invasive treatments that reduce patient discomfort are important. Traditionally, TFA is the main method used in TACE. However, TFA angiography requires long-term compression of the groin area, limited flexion of the lower limbs, and rest, which causes back pain and other discomfort for patients. In contrast, TRA does not require manual pressure to stop bleeding after sheath removal, and patients can return to the room in a wheelchair after surgery, do not need to lie down, and can sit and walk. TRA has become the mainstream in the cardiovascular field, and the risk of developing cerebrovascular complications is comparable to that of TFA. Further, some reports have shown that TRA is safe [3,9,10].

In this study, we conducted abdominal angiography and provided treatment for TRA in 1601 patients at multiple centers. Anatomically, there are no major nerves running around the radial artery, thereby making it a relatively safe site for puncture. Moreover, the incidence rate of nerve damage and hematoma formation is low [8]. There are some cases in which the arteries are narrow and challenging to palpate. Hence, puncturing is challenging to perform. However, puncturing becomes easier under ultrasound guidance [11]. It takes some time for the sheath to be placed in the radial artery and passed through the aortic arch and to obtain the first contrast image of the celiac artery. The reason for this is that there are cases in which a pigtail catheter is required to cannulate the descending aorta because of the shape of the aortic arch. In such cases, the catheter must be replaced. The treatment success rate was high, and the length of hospital stay was similar to that previously reported for TFA. Moreover, the incidence of complications specific to TRA was low [12-14]. Three cases of serious TRA-related complications were observed. Notably, all these complications occurred during catheter cannulation from the left subclavian artery to the descending aorta. Kawamura et al. performed a preoperative CT scan and classified the left subclavian artery into three patterns based on the anatomical shape of the aortic arch and the location of the left subclavian artery bifurcation. Therefore, it is possible to predict in advance the degree of difficulty in cannulating the descending aorta and decrease the risk of RAVI-related cerebrovascular complications [7]. When passing through the aortic arch, it is important to cautiously advance the catheter under guide wire guidance, thereby not spending too much time manipulating the aortic arch, change to a senior physician, and use a pigtail catheter if necessary.

According to the number of years of RAVI experience, operators with <1 year of experience had a significantly longer time from sheath placement to the first contrast imaging. However, no difference was observed in the procedural success rate or complication rate. Toyoda et al. reported that this technique can be learned in a year [6]. Patients aged 80 years had a history of and a higher risk for cardiovascular events. However, there were no significant differences in procedure time, procedural success rate, and complication rate between patients aged <80 years and those aged ≥80 years). Cannulation of the celiac artery and superior mesenteric artery using TRA could be performed and stable even in cases in which TFA was difficult due to curvature of the abdominal aorta caused by arteriosclerosis or in subtypes where the common or right hepatic artery branches from the superior mesenteric artery. In terms of the disadvantages of RAVI, if the communication between the ulnar and radial arteries is poor (negative Allen's test) [15], or if the radial artery must be preserved for shunting in hemodialysis patients or those planning to transition to dialysis, TRA is not indicated. In addition, left radial access is difficult if the HCC receives nourishment from the right internal mammary artery.

The current study had several limitations. For example, it was retrospective in nature. The devices and equipment used were different because the study was performed at multiple centers. Moreover, only data on TRA were used. Therefore, to validate the results of this study, a multicenter prospective cohort study comparing TRA and TFA should be conducted.

## Conclusions

Hepatic angiography from the radial artery and TACE can be used more widely in the future due to their safety and reliability, which are similar to those of TFA. Moreover, these approaches can reduce patient burden and improve the quality of treatment.

## References

[1] Heimbach JK, Kulik LM, Finn RS (2018). AASLD guidelines for the treatment of hepatocellular carcinoma. Hepatology.

[2] Kudo M, Kawamura Y, Hasegawa K (2021). Management of hepatocellular carcinoma in Japan: JSH consensus statements and recommendations 2021 Update. Liver Cancer.

[3] Brueck M, Bandorski D, Kramer W, Wieczorek M, Höltgen R, Tillmanns H (2009). A randomized comparison of transradial versus transfemoral approach for coronary angiography and angioplasty. JACC Cardiovasc Interv.

[4] You K, Guo T, Sun D, Song H, Liu Z (2023). Transradial versus transfemoral approach for TACE: a retrospective study. BMC Gastroenterol.

[5] Minici R, Serra R, Giurdanella M, Talarico M, Siciliano MA, Carrafiello G, Laganà D (2023). Efficacy and safety of distal radial access for transcatheter arterial chemoembolization (TACE) of the liver. J Pers Med.

[6] Toyoda H, Yasuda S, Shiota S (2021). Safety, feasibility, and comfort of hepatic angiography and transarterial intervention with radial access for hepatocellular carcinoma. JGH Open.

[7] Kawamura Y, Akuta N, Fujiyama S (2023). A new imaging classification for safer radial access visceral intervention of the liver and optimal case selection: a preliminary report. Hepatol Res.

[8] Shiozawa S, Tsuchiya A, Endo S (2003). Transradial approach for transcatheter arterial chemoembolization in patients with hepatocellular carcinoma: comparison with conventional transfemoral approach. J Clin Gastroenterol.

[9] Sanjit S Jolly, Salim Yusuf, John Cairns (2011). Radial versus femoral access for coronary angiography and intervention in patients with acute coronary syndromes (RIVAL): a randomised, parallel group, multicentre trial. Lancet.

[10] Völz S, Angerås O, Koul S (2019). Radial versus femoral access in patients with acute coronary syndrome undergoing invasive management: a prespecified subgroup analysis from VALIDATE-SWEDEHEART. Eur Heart J Acute Cardiovasc Care.

[11] Miller AG, Bardin AJ (2016). Review of ultrasound-guided radial artery catheter placement. Respir Care.

[12] Zhang X, Luo Y, Tsauo J (2022). Transradial versus transfemoral access without closure device for transarterial chemoembolization in patients with hepatocellular carcinoma: a randomized trial. Eur Radiol.

[13] Yamada R, Bracewell S, Bassaco B (2018). Transradial versus transfemoral arterial access in liver cancer embolization: randomized trial to assess patient satisfaction. J Vasc Interv Radiol.

[14] Hedjoudje M, Barat M, Dohan A (2024). Comparison between radial and femoral artery access for transarterial chemoembolisation in patients with hepatocellular carcinoma. Can Assoc Radiol J.

[15] Valgimigli M, Campo G, Penzo C, Tebaldi M, Biscaglia S, Ferrari R (2014). Transradial coronary catheterization and intervention across the whole spectrum of Allen test results. J Am Coll Cardiol.

